# The relationship between distributed leadership and the enthusiasm and professional collaboration levels of teachers in Türkiye: the moderator effect of teacher optimism

**DOI:** 10.3389/fpsyg.2026.1729734

**Published:** 2026-03-04

**Authors:** Yurdagül Doğuş

**Affiliations:** Department of Educational Administration, Faculty of Education, Kocaeli University, Kocaeli, Türkiye

**Keywords:** distributed leadership, moderated mediation, teacher collaboration, teacher optimism, teaching enthusiasm

## Abstract

**Introduction:**

The professional collaboration of teachers is highly important for the success of educational outcomes. This study aimed to determine the conditions under which distributed leadership influenced the collaboration of teachers. The study focused on identifying the previously undetermined complex relationships between distributive leadership, teacher professional collaboration, teaching enthusiasm, and teacher optimism, which were found in the literature to be associated with improving the quality of education, enhancing teaching, and achieving successful student outcomes.

**Methods:**

Data collected from 547 teachers working at 38 different public schools in 20 provinces across Türkiye were analyzed in a cross-sectional manner using the bootstrap method. The effects of distributed leadership on teacher collaboration were examined using a moderated mediation model in which teaching enthusiasm was the mediator variable, and teacher optimism was the moderator variable.

**Results:**

The results revealed that distributed leadership had direct and indirect significant effects on teacher collaboration through the mediation of teaching enthusiasm. Additionally, teacher optimism plays a moderating role in both the effect of distributed leadership on teaching enthusiasm and the effect of distributed leadership on teacher collaboration through teaching enthusiasm. In other words, the positive effects of distributed leadership became stronger in cases where teacher optimism levels were high.

**Discussion:**

The leadership approach which distributes positive emotions such as optimism and enthusiasm, which are at the core of positive psychology, and leadership roles to all teachers in the school, can help increase collaboration among teachers and positive workplace behaviors. This research indicates that teachers being enthusiastic while teaching and taking an active role in school management may enable them to be more collaborative in fulfilling these roles and other teaching roles. Teachers’ enthusiasm for teaching can function as an emotional mechanism that connects teachers’ collaboration with a leadership approach that distributes leadership throughout the school. Moreover, when teachers are more optimistic, they trust school administrators’ leadership practices more, participate more in management, and can teach more collaboratively and enthusiastically. Future research may further examine these associations across different cultural contexts.

## 1 Introduction

The world of science acknowledges the determining roles of teacher qualifications and school leadership in the quality of education and the success of students (Darling-Hammond, 2000; Kasalak and Dağyar, 2020). Due to its effectiveness in the improvement of teacher qualifications, collaboration among teachers has been the focus of researchers for more than half a century (Epperson, 1962; Hargreaves and O'Connor, 2018; Xie et al., 2023). Additionally, every passing day, new evidence is added to the knowledge base about the contributions of the emotional characteristics of teachers to their professional relationships and the learning of their students (Dong and Xu, 2022; Lambersky, 2016; Ma, 2023; Namaziandost et al., 2024). However, the effects of the emotional characteristics of teachers on the relationship between the professional collaboration of teachers (TPC) and a leadership approach that distributes leadership to the entirety of the school are still unclear (Honingh and Hooge, 2014; Karaköse et al., 2022; Keller et al., 2016; OECD, 2019a). Therefore, to improve teaching processes, it is important to focus on the interactions between TPC and distributed leadership (DL), as well as emotional characteristics such as teacher optimism (TO) and teaching enthusiasm (TE).

Schools differ from other organizations due to their unique culture and values regarding human relations, and they directly influence the entire society. This is because schools are a small model of the society in which they exist, and all members of a society interact directly with schools for a significant part of their lives, whether in the roles of student, teacher, administrator, or employee, or through various collaborative and commercial activities (Bickmore, 2011). Today, expectations regarding schools go far beyond their traditional roles. All segments of society now expect schools to educate students in a multifaceted way. Therefore, it does not seem possible for schools, which have extremely chaotic human relations, academic problems, and an economic structure that needs to be managed, to be run solely by “heroic school leaders” (Hulpia and Devos, 2010; Spillane, 2005). For schools to meet the expectations placed upon them, it is necessary to take into account the school’s culture, human relations, functioning, teachers’ emotions, and values with a positive approach. In addition, it is almost imperative that school administrators have the understanding and competence to bring teachers together around shared goals, distribute leadership roles to teachers to achieve these goals, strengthen cooperation among teachers, and create a school environment that enables teachers to teach with enthusiasm and students to learn fully (Hoy and Tarter, 2011; Terjesen et al., 2004; Vangrieken et al., 2015).

TPC is considered fundamental to the development of teachers and the success of students and is deemed necessary by international organizations (OECD, 2014, 2019a, 2019b). For example, professional collaboration is one of the 12 standards for training qualified personnel in the National Staff Development Scheme of the United States of America (Kolleck et al., 2020). In addition to this, researchers have pointed to several positive organizational and individual consequences of TPC, especially its effects on student outcomes (Duyar et al., 2013). For instance, when teachers collaborate with their colleagues, they can use innovative pedagogies more in their classes (Büyükgöze et al., 2024; OECD, 2014), and this can affect the success of students positively (Egodawatte et al., 2011). Moreover, researchers have emphasized the relationships between TPC and the motivation of teachers (Durksen et al., 2017; Reeves et al., 2017), their job satisfaction levels (Kolleck et al., 2020; OECD, 2014), and their professional learning activities (Durksen et al., 2017).

TPC is influenced by multiple factors (Horn and Little, 2010). In a comprehensive study, Kolleck et al. (2020) found that one of the important factors encouraging teachers to collaborate was leadership practices. In other words, professional collaboration among teachers increases when their administrators support them, promote their participation in decision-making, and provide them with resources (Honingh and Hooge, 2014; Sheppard et al., 2010). These desirable characteristics of school administrators remind us of the fundamental and strong aspects of DL, which distributes leadership roles to other stakeholders of the school (Spillane, 2005). The relevant literature has shown that the distribution of leadership responsibilities to stakeholders has positive outcomes in terms of school administrators, teachers, and the success of students (Bush and Glover, 2012; Büyükgöze et al., 2024; Karaköse et al., 2022). At these collaborative schools where leadership is distributed, teachers can develop more positive attitudes toward their profession and school (Liu and Printy, 2017). This is why it is considered a practical necessity for education systems to distribute leadership to the entire school and ensure collaboration among teachers (Lin, 2022).

Some emotional characteristics of teachers also have a determining role in TPC (Kolleck et al., 2020). Two of these characteristics are enthusiasm and optimism (Dong and Xu, 2022; Öngel and Tabancalı, 2022). TE is seen as one of the basic requirements for the motivation and success of students (Burić, 2019; Kunter et al., 2011). The optimism of teachers allows them to have a positive attitude toward the profession and life (Seligman, 2020). Optimistic teachers take responsibility, turn challenges into opportunities, and collaborate effectively with all stakeholders for the success of students (Dong and Xu, 2022). Furthermore, in the profession of teaching, which is constantly becoming more difficult, the professional enthusiasm and optimism of teachers can make collaboration at schools with DL practice even stronger (Dong and Xu, 2022).

Individuals have fundamental motivational needs such as competence, autonomy, and relationship building (Deci and Ryan, 2008). Individuals want to make their own decisions and initiate their own behaviors. They want to use their abilities and overcome the challenges they face with these abilities (Bandura, 2001). They want their achievements to be recognized by those around them and to be accepted as a valued member of a group. Teachers are no exception. Schools are both a workplace environment for teachers and a social environment due to the close relationships within the teachers’ lounge (Deci and Ryan, 2000). When school administrators provide teachers with autonomy in their profession, facilitate collaboration with colleagues, and give them high-level responsibilities related to school management-in other words, when they exhibit DL characteristics-teachers can satisfy a significant portion of their needs for competence, autonomy, and relatedness (Deci and Ryan, 2008; Spillane, 2005; Bennett et al., 2003). Thus, their perceptions of self-efficacy can be strengthened, and they can feel valued by their principals (Bandura, 1982). School administrators strengthen teachers’ skills such as leadership and innovation by distributing leadership (Torres, 2019). Teachers’ ability to carry out their own projects and pedagogical practices and to change school practices through joint decisions can make them feel valued by their administrators. Teachers who feel valued by their administrators may have higher intrinsic motivation and deliver more enthusiastic teaching (Burić and Moè, 2020; Deci and Ryan, 2000).

DL has been empirically studied mostly in Western countries. Evidence regarding whether DL is also valid in different cultural contexts is limited (Adams et al., 2025; Bolden, 2011; Printy and Liu, 2020). This is why, to understand the relationship between DL and TPC better, it is necessary to refer to findings obtained in non-Western educational contexts (Mifsud, 2023). This study, which examines the relationship between DL and TPC in the context of teachers’ emotional characteristics such as TE and TO, based on the responses of Turkish teachers in face-to-face education processes and formal education practices in schools, may provide more comprehensive findings.

The study expands previous studies in three main dimensions. First, the TPC and the DL-related characteristics of school administrators were focused on. TPC is a teacher quality that is frequently highlighted in the process of increasing student success (Goddard et al., 2007; Honingh and Hooge, 2014). In this context, Kolleck et al. (2020) argued that TPC has not been investigated enough using theoretical approaches and validated scales, whereas Honingh and Hooge (2014) asserted that TPC has usually been discussed as a mediator variable in research, and thus, there is a lack of sufficient information related to TPC. This study contributes to the literature by examining TPC as the dependent variable and using validated scales.

Second of all, the view that the democratic structure of DL plays an important role in improving not only the capacities of teachers but also the learning of students by promoting TPC is constantly gaining more support (Karaköse et al., 2022). Some researchers (Liu S. et al., 2021) also argued that there was not enough empirical evidence in the literature about DL yet, and the academic interest in DL remains. Similarly, in the literature, there has been an emphasis on the need to discover the opportunities and limits of DL by conducting more studies on it (Harris, 2009; Sheppard et al., 2010) and examine how DL contributes to organizational performance (Leithwood et al., 2009). Although the outcomes of DL have been investigated in different contexts by utilizing data from international comparisons, especially PISA and TALIS (Büyükgöze et al., 2024; Liu Y. et al., 2016; Çoban and Atasoy, 2020), there is still little known about the direct and indirect implications of the emotional characteristics of teachers such as TE and TO in the relationship between DL and TPC (Honingh and Hooge, 2014; Sheppard et al., 2010). What is more, it is understood that there is a gap in the literature regarding the mediator role of TE and the moderator role of TO in the relationship between DL and TPC. In this study, it was aimed to contribute to the literature by offering empirical evidence in this relatively less investigated field.

Third, there is criticism in the literature that very few studies have been conducted on TE (Trauernicht and Lazarides, 2024). Bardach et al. (2022) recommended that there be more focus on the enthusiasm experienced by teachers, while according to Kunter et al. (2011), the indirect relationships concerning TE should be uncovered, and the professional behaviors influential on TE should be identified. In their comprehensive study investigating TE, Keller et al. (2016) underlined that future studies need to discuss the emotional experiences associated with the pleasure and enthusiasm felt by teachers while teaching. In this context, while examining the mediator role of teaching enthusiasm in the relationship between DL and TPC, this study demonstrates the moderator effect of TO in this relationship and contributes to the literature. In this process, answers to the following research questions are presented:

1. Does teaching enthusiasm have a mediator effect in the relationship between distributed leadership and the professional collaboration of teachers?
2. Does teacher optimism have a moderator role in the effect of distributed leadership on teaching enthusiasm?
3. Does teacher optimism have a moderator role in the indirect effect of distributed leadership on the professional collaboration of teachers mediated by teaching enthusiasm?

### 1.1 Context of Turkish culture and Turkish education system

The leadership approaches of school principals and the behaviors of teachers are shaped by cultural variables (Kılınç and Özdemir, 2025). Turkish culture has a collaborative aspect that is known as “imece,” which can be roughly translated as “collective work.” With the additional effect of the Islamic belief, in Türkiye, most teachers consider teaching knowledge to other people to be a sacred deed (Budak and Kula, 2017). This, in turn, may lead teachers to be more enthusiastic about teaching (Öngel and Tabancalı, 2022). While it is not easy to describe the extent to which leadership is distributed in Türkiye, which has a highly centralized education system (Özdemir and Demircioğlu, 2014), there are educational policies that aim at distributing leadership. For example, there are directives requiring teachers of a course, a certain classroom, or special needs students to perform their duties in collaboration with their colleagues (MoNE, 2013). “Educational Vision for 2023”, one of the important policy documents in recent years, places special emphasis on the collaboration between teachers and their colleagues (MoNE, 2018). In addition to this, although the Turkish education system is associated with a high level of power distance, policy documents include DL practices. For example, school principals delegate the budget to parent-teacher associations, they transfer administrative duties to vice principals, and decisions are made in collaboration in meetings of councils of teachers (MoNE, 2013). It was stated that keeping DL behaviors in mind, especially in transformation and restructuring processes, is functional (Özer and Beycioğlu, 2013). In this sense, in the success of the Türkiye Yüzyılı Maarif Modeli [Educational Model for the Century of Türkiye] reform initiative started in 2024 and expected to pioneer the second century of the Republic of Türkiye, the DL roles of principals can be determining.

### 1.2 Theoretical framework and hypotheses

**Figure 1:**
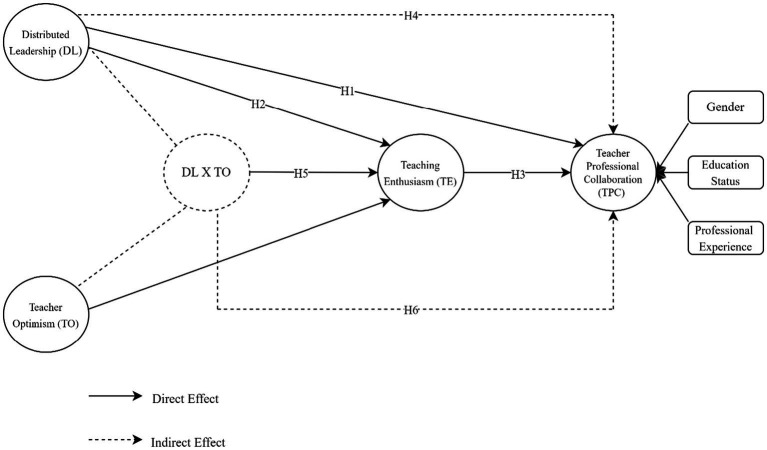
Conceptual model of the study.

This study is founded upon the Positive Psychology Approach and Distributed Leadership Theory with the evidence it provides in the context of Türkiye. In 1998, positive psychology changed the course of the science of psychology with the call of Martin Seligman to build a flourishing life, and it has developed as an approach that focuses on happiness, well-being, and positive experiences (Seligman, 2019). DL theory is a relatively recent approach that does not reduce leadership to individual characteristics, instead, it is based on activity theory and distributed consciousness and encompasses leadership as a process involving the collective and the stakeholders (Gronn, 2000; Karaköse et al., 2022). DL theory, which characterizes leadership with transformative, collaborative, interactive, participative, fluid, dynamic, and humane characteristics, is also compatible with the principles of positive psychology in this respect (Adams et al., 2025; Harris, 2009; Spillane, 2005). In line with the evidence offered by the current literature, this study involved DL to capture the leadership aspect, TPC to capture the relationship aspect, and TE and TO to capture the emotional aspect. This study mainly focused on the moderator role of teacher optimism in the relationships between DL in Türkiye and TPC and teaching enthusiasm. This study shows that the DL approach, which empowers teachers by giving them responsibility and autonomy, further stimulates their TE, and that teachers with increased TE perform their profession more passionately and are more willing to collaborate with their colleagues. In the relationship between DL and TPC, the mediation of TE increases when TO increases and decreases when TO weakens. The research provides a significant theoretical contribution to the literature by demonstrating that TO shapes this mechanism (the mediation of TE in the relationship between DL and TPC). It offers evidence of what strengthens the relationship between DL and TPC. The research expands the knowledge base showing that leadership practices in schools affect teachers’ emotions, and that teachers’ emotions, in turn, affect the organizational functioning of the school. The conceptual model and hypotheses of the study are presented in Figure 1.

#### 1.2.1 Professional collaboration of teachers

TPC, which has meanings beyond a simple collaboration, may refer to complex patterns varying from the work carried out by teachers of a particular course by gathering to collective collaboration extending to the entire school (Slavit et al., 2011). This way, as the collaborative work carried out by teachers affects the learning of students positively (Reeves et al., 2017), it may also be effective in increasing their comfort, optimism, and enthusiasm while performing their duties by helping them establish positive interpersonal relationships (Bardach et al., 2022; Kolleck et al., 2020). In this study, TPC was defined as a context in which teachers share their teaching strategies, take good practices as examples, and solve problems together to improve the learning of students (Demir, 2014). To conduct these collaborative activities, teachers need support (Slavit et al., 2011). Teachers can receive this support from DL, which strengthens their leadership skills and collaborative work.

#### 1.2.2 Distributed leadership

DL has made its mark in the last quarter century within leadership research (Gümüş et al., 2018). Spillane (2005) defined DL as a system of practice consisting of a leader, followers, and situation. In addition to this, DL is frequently described as a group dynamic functioning within and through relationships (Bennett et al., 2003), a process in which leadership is shared (Bolden, 2011), and a characteristic of the capacities of a school (Liu Y. et al., 2021). In this study, DL was considered a form of leadership in which principals collaborated with all stakeholders, involved these stakeholders in decision-making and problem-solving processes, and promoted mutual support and cooperation (Özer and Beycioğlu, 2013).

Teachers have a wide variety of duties and responsibilities within their daily routines, including determining the curriculum, teaching students the curriculum’s learning outcomes, assessing what has been taught, teaching in teams, observing other colleagues’ lessons, forming professional networks, conducting observation visits at different schools, and participating in professional learning activities (Hargreaves and O’Connor, 2018; Honingh and Hooge, 2014; OECD, 2014; Reeves et al., 2017; Vangrieken et al., 2015). Teachers need to collaborate with each other to fulfill these duties and responsibilities. In this process, while teachers collaborate with their colleagues, they need the DL characteristics of their principals that prioritize relationships and collaboration. Because DL, through the multifaceted mechanisms they employ, encourages teachers to take on decision-making roles in certain important school matters, collaborate with their colleagues, and foster an inclusive school environment (Hulpia et al., 2009; Liu Y. et al., 2021). DL applications place great importance on establishing personal interaction with teachers and continuing their professional development (Harris, 2008; Hulpia and Devos, 2010). DL facilitates greater collaboration by increasing teachers’ job satisfaction (Kılınç and Özdemir, 2025; Liu Y. et al., 2021) and organizational commitment (Aboudahr and Jiali, 2019). Similarly, it was emphasized that the involvement of teachers in decision-making processes by DL increased the collaboration among teachers (Sheppard et al., 2010). Based on the results of different studies that have identified the relationship between DL and TPC (Çoban and Atasoy, 2020; Karaköse et al., 2022; Lin, 2022; Ma and Marion, 2024), in this study, it was also expected a direct and positive relationship between DL and TPC. Accordingly, hypothesis H1 was as follows:

*H1*: There is a positive significant relationship between distributed leadership and the professional collaboration of teachers.

#### 1.2.3 Teaching enthusiasm

As an emotional trait of teachers, enthusiasm has been conceptualized in the literature as teacher enthusiasm (Keller et al., 2016). There are two classifications of teacher enthusiasm. In the first classification, teacher enthusiasm was conceptualized as a two-dimensional construct involving subject enthusiasm and teaching enthusiasm (Kunter et al., 2011). In the second classification, it was also considered a two-dimensional construct, this time consisting of experienced enthusiasm and observed enthusiasm (Keller et al., 2016). In this study, the first classification was adopted and the focus was on the dimension of teaching enthusiasm within teacher enthusiasm. TE is considered an emotional orientation that reflects the pleasure taken from the teaching process and is associated with the motivation of the teacher (Mahler et al., 2017). Hence, in this study, as defined by Kunter et al. (2011), TE was discussed as an emotional and individual trait that is materialized through subjective experiences such as pleasure, excitement, and delight and some behaviors displayed in the classroom.

TE, a characteristic related to teachers’ enjoyment of teaching, their intrinsic motivation, and their emotional orientation, can become stronger in schools managed with the DL approach (Deci and Ryan, 2000; Kunter et al., 2011). Because DL understanding contributes to the creation of a positive and supportive environment at school, it makes teachers’ perceptions and feelings about their profession more positive (Zhang et al., 2025). Teachers who lack enthusiasm for their profession may not produce successful results and may decide to leave the teaching profession (Lambersky, 2016). Lack of enthusiasm among teachers is one of the signs of burnout (Kasalak and Dağyar, 2020). A leadership approach that involves teachers in decision-making processes (Harris et al., 2007), grants them autonomy in their profession, and prioritizes shared responsibility and a collaborative school culture develops teachers’ competencies in different areas and empowers them (Torres, 2019). It enables teachers to find their profession more meaningful, feel more satisfaction from teaching (Liu Y. et al., 2021), and be more committed to their schools (Aboudahr and Jiali, 2019). Teachers’ enjoyment of their profession and their commitment to their schools are important organizational and emotional mechanisms that enable them to teach with greater enthusiasm. This is because TE is an emotional structure shaped by teachers’ commitment to their profession.

Teachers with high commitment can deliver more enthusiastic teaching (Leithwood and Beatty, 2008). Some studies have demonstrated the relationship between TE and the motivation of teachers (Kunter et al., 2011). Accordingly, based on the information in the relevant literature, a positive relationship between DL and teaching enthusiasm was anticipated and this anticipation was tested through hypothesis H2.

*H2*: There is a positive significant relationship between distributed leadership and teaching enthusiasm.

#### 1.2.4 Relationship between teaching enthusiasm and the professional collaboration of teachers

TE, which is explained by the intrinsic values of teachers and the pleasure they feel, is accepted as a vivid and interesting teaching behavior, an effective teaching strategy, and a teacher trait associated with teaching skills (Kunter et al., 2011). Researchers have listed some of the typical characteristics of teachers with TE as being softspoken, using facial expressions, making eye contact, moving while teaching, and smiling (Kunter et al., 2011; Murray, 1983). These characteristics are very similar to those of individuals who have collaborative tendencies (Johnston et al., 2010). TE is one of the requirements of quality education. TE is an indicator of teachers’ motivation toward their profession and the effort they expend for their students’ success (Kunter et al., 2013). Teachers who possess TE can contribute to the formation of a collaborative culture in their schools through certain emotional and motivational pathways. For example, teachers with high TE can spread their enthusiasm to their colleagues during daily school activities (Hatfield et al., 1993). Positive emotions such as TE enhance the development of individuals’ social and psychological resources and encourage new ideas and actions (Hobfoll, 2002). The commitment of teachers with TE to their profession can be a source of motivation for other teachers (Burić and Moè, 2020). Thus, teachers who are synchronized in TE can become more consistent in their behavior and engage in more collaboration (Herrando and Constantinides, 2021).

When teachers collaborate with their colleagues, they share experiences that can improve the quality of education and enhance student learning (Goddard et al., 2007; Reeves et al., 2017). Teachers who collaborate with their colleagues can also work together to find solutions to problems encountered at school and in the classroom, share the workload, and feel less isolated (Vangrieken et al., 2015). This can make these teachers more energetic and more enthusiastic and passionate about teaching. In addition, in their study examining the relationship between TE and collaborative school cultures, Öngel and Tabancalı (2022) found a positive relationship between teaching enthusiasm and collaborative teacher behaviors. Based on the information in the literature, a positive relationship between TE and TPC was expected. Accordingly, hypothesis H3 was as follows:

*H3*: There is a positive significant relationship between teaching enthusiasm and the professional collaboration of teachers.

#### 1.2.5 Mediator role of teaching enthusiasm in the relationship between distributed leadership and the professional collaboration of teachers

Previous studies have emphasized that school principals with a DL approach involve teachers and other stakeholders of the school in decision-making processes and show collaborative efforts (Nadeem, 2024). DL not only increase the capacity of the school but also improve the leadership skills and collaboration of teachers by utilizing the expertise and insights of all stakeholders (Printy and Liu, 2020). Therefore, one may argue that enthusiastic teachers may be particularly more willing to collaborate with their colleagues at schools where they can participate in administrative processes. Besides, the current literature has demonstrated the relationships between TE and DL (Öngel and Tabancalı, 2022) and between DL and TPC (Çoban and Atasoy, 2020; Karaköse et al., 2022; Ma and Marion, 2024). Based on the current literature, as well as other hypotheses (H1 and H3), a connection between DL and TPC through the mediation of TE was expected. Accordingly, hypothesis H4 was as follows:

*H4*: Teaching enthusiasm mediates the relationship between distributed leadership and the professional collaboration of teachers.

#### 1.2.6 Teacher optimism

Optimism, which is one of the important constructs proposed by positive psychology, reflects the positive attitudes possessed by individuals toward themselves, others, and life (Seligman, 2020). In this study, as described in the context of teacher agency by Liu S. et al. (2016), TO was considered in the context of the possession of a generally optimistic personality by teachers. According to this definition, TO refers to the establishment of good communication and positive relationships by teachers with their colleagues, their belief that they will face good things, and their optimistic points of view regarding the future (Liu S. et al., 2016).

As previously mentioned, schools are chaotic and difficult organizations to manage. Administrators and teachers encounter unexpected situations in management and teaching processes, take on challenging tasks, and experience stressful times (Herman et al., 2018). Optimism, one of teachers’ emotional assets, plays an important role in helping them cope with the challenges of school life (Borralho et al., 2025; Merino-Tejedor et al., 2020; Yang, 2022). Furthermore, teachers’ optimism is one of the determinants of how they interpret school administrators’ leadership practices and how they feel about these practices (Jennings and Greenberg, 2009). Optimistic teachers focus on the positive aspects of their administrators, view challenges as temporary situations, and have a positive attitude toward the leadership practices demonstrated by their administrators (Schueller and Seligman, 2008; Seligman, 2020). In short, optimistic teachers do their best to perform educational activities in an excellent manner by neglecting negativity (Dong and Xu, 2022). This is why the optimistic tendencies of teachers may moderate the relationship between the distribution of leadership by principals and the enthusiasm of teachers to teach. The evidence in the literature suggests that TPC is influenced by not only organizational characteristics like leadership but also the individual characteristics of teachers like TE (Honingh and Hooge, 2014; Ma and Marion, 2024). Based on this evidence, it was expected that the optimism levels of teachers could affect the relationship between DL and TE and established the following hypothesis:

*H5*: Teacher optimism moderates the relationship between distributed leadership and teaching enthusiasm.

Additionally, in the study, based on hypotheses H4 and H5 and previous studies proposing indirect connections between DL and TPC (Liu Y. et al., 2021), it was expected that teacher optimism would have a moderator role in the indirect effect of DL on TPC mediated by TE. The final hypothesis, which was created based on this expectation, was as follows:

*H6*: Teacher optimism moderates the indirect effect of distributed leadership on the professional collaboration of teachers mediated by teaching enthusiasm.

## 2 Method

This study was planned with a quantitative approach and conducted with a cross-sectional design by collecting data within a specific time period.

### 2.1 Participants and data collection

The sample of the study consists of teachers working in western, eastern, northern, and southern cities of Turkey. In order to reflect the socio-economic and cultural characteristics of Turkey, the sample group was formed of teachers working in cities from different regions. The data for the study was collected after obtaining ethical approval. Data were collected from 20 cities and 38 public schools across Turkey through convenience sampling by contacting school principals via email and telephone. School principals were provided with detailed information about the study and asked for their support in data collection. A voluntary consent form was added to the online scale form. The online scale form was sent only to teachers working in the relevant schools via the WhatsApp application. No other communication channel was used during the data collection process; all participants were reached using the same method. Therefore, there was no need to test for possible response differences arising from the data collection method. The online scale form consisted of questions about the school (school type) and the teacher (gender, educational status, and professional seniority), as well as 28 scale items. Anonymity was ensured by not requesting any identifying information about the participants in the online form. Participants were also asked to complete the scale questions on a voluntary basis. A total of 750 participants from 38 schools were reached, however, 563 participants responded to the scales. The responses of 16 participants were removed from the datasets because they contained outlier values. This way, the data of 547 participants were used in the analyses, and a return rate of 72% was achieved. In this regard, 53.7% (*n* = 294) of the teachers participating in the study were male, and 46.3% (*n* = 253) were female. The vast majority of teachers were bachelor’s degree holders (84.8%; *n* = 464), while 15.2% (*n* = 83) had graduate education. When examining the school level at which they work, 23.2% of teachers (*n* = 127) work in preschool, 28.3% (*n* = 155) in elementary school, 22.3% (*n* = 122) in middle school, and 26.1% (*n* = 143) in high school. The average professional seniority of the participating teachers is 13.76 years (*SD* = 10.98) (see Table 1).

**Table 1:** Demographic characteristics of participants (*n* = 547).

Variables	Categories	Mean (SD)	*n*	%
Gender	Male		294	53.7
	Female		253	46.3
Educational status	Bachelor’s Degree		464	84.8
	Graduate Degree		83	15.2
Level of the school worked at	Preschool		127	23.2
	Elementary School		155	28.3
	Middle School		122	22.3
	High School		143	26.1
Professional seniority		13.76 (10.98)		

*n*, number of participants; SD, standard deviation.

### 2.2 Variables and data collection instruments

When determining the measurement tools used in the data collection process of the study, the linguistic and cultural equivalence of the items was taken into consideration. To prevent conceptual confusion with the original scales and to ensure measurement reliability, the Turkish versions of the scales, which had previously undergone validity and reliability studies on a Turkish sample and had their psychometric properties verified, were used instead of the original forms of the scales in this study. In this context, detailed information about the scales used in the study is presented below.

*Independent (Predictor)* Var*iable: Distributed Leadership Scale (DLS)*. The DL behaviors of school principals were measured using DLS, which is a unidimensional scale consisting of 10 items (item example: the administrators of our school make efforts to create a cooperative school environment) developed by Özer and Beycioğlu (2013). DLS is a 5-point Likert-type scale in which each item has response options varying from 1 (never) to 5 (always). The confirmatory factor analysis (CFA) of the scale conducted in this study showed acceptable goodness-of-fit values (*χ*^2^/df = 125.329/35 = 3.58; RMSEA = 0.069, SRMR = 0.012, CFI = 0.98, and TLI = 0.98). The Cronbach’s alpha and composite reliability (CR) coefficients of the scale were 0.97, and its average variance extracted (AVE) value was 0.81. Furthermore, since the square root of DLS’s AVE value (√AVE = 0.90) is higher than the correlations with the structures of the TE (*r* = 0.39), TO (*r* = 0.50), and TPC (*r* = 0.38) scales, the discriminant validity of DLS has been ensured according to the Fornell and Larcker (1981) criterion (see Table 2).

**Table 2:** Results of Pearson correlation analysis between variables with mean, standard deviation, AVE, √AVE, Cronbach alpha, and CR values (*n* = 547).

Variables	*M*	*SD*	AVE	√AVE	*α*	CR	DLS	TES	TOS	TPCL
DLS	4.26	0.76	0.81	0.90	0.97	0.97	—			
TES	3.41	1.08	0.61	0.78	0.86	0.88	0.39^**^	—		
TOS	4.07	0.77	0.76	0.87	0.94	0.94	0.50^**^	0.66^**^	—	
TPCS	3.29	0.73	0.53	0.73	0.75	0.75	0.38^**^	0.52^**^	0.54^**^	—
Gender	—	—	—	—	—	—	−0.02	−0.16^**^	−0.11^*^	−0.08^*^
Educational status	—	—	—	—	—	—	0.05	−0.00	−0.03	0.04
Professional seniority	13.76	10.98	—	—	—	—	0.02	0.03	0.01	0.05

*p < 0.05; **p < 0.01; DLS, distributed leadership scale; TES, teaching enthusiasm scale; TOS, teacher optimism scale; TPCS, teacher professional collaboration scale; M, mean; SD, standard deviation; AVE, average variance extracted; √AVE, square root of value AVE; α, Cronbach alpha; CR, composite reliability; Gender: The reference group is male; Educational status: The reference group has a bachelor’s degree.

*Mediator Variable: Teaching Enthusiasm Scale (TES).* Teachers’ teaching enthusiasm was measured using the 5-item Teaching Enthusiasm subscale of the Teacher Enthusiasm Scale developed by Kunter et al. (2011) (example item: I enjoy teaching my class). The scale was adapted to Turkish by Kasalak and Dağyar (2020). TES is a 5-point Likert-type scale in which each item has response options varying from 1 (absolutely disagree) to 5 (absolutely agree). The CFA of the scale conducted in this study showed high goodness-of-fit values (*χ*^2^/df = 2.221/4 = 0.55; RMSEA = 0.000, SRMR = 0.005, CFI = 1.00, and TLI = 1.00). The Cronbach’s alpha, CR, and AVE values of the scale were calculated as 0.86, 0.88, and 0.61, respectively. In addition, since the square root of the AVE value of TES (√AVE = 0.78) is higher than the correlations with the structures of DL (*r* = 0.39), TO (*r* = 0.66), and TPC (*r* = 0.52), according to the Fornell and Larcker (1981) criterion, the TES has discriminant validity (see Table 2).

*Moderator Variable: Teacher Optimism Scale (TOS).* Teachers’ levels of optimism were determined using the 5-item Teacher Optimism Subscale of the Teacher Agency Scale developed by Liu S. et al. (2016) and adapted into Turkish by Bellibaş et al. (2019) (sample item: I am optimistic about my future). TOS is a 5-point Likert-type scale in which each item has response options varying from 1 (absolutely disagree) to 5 (absolutely agree). The CFA of the scale conducted in this study showed high goodness-of-fit values (*χ*^2^/df = 3.813/3 = 1.27; RMSEA = 0.022, SRMR = 0.004, CFI = 1.00, and TLI = 0.99). The Cronbach’s alpha and CR coefficients of the scale were 0.94, and its AVE value was 0.76. On the other hand, since the square root of the AVE value of the TOS (√AVE = 0.87) is higher than the correlations with the structures of DL (*r* = 0.50), TE (*r* = 0.66), and TPC (*r* = 0.54), the discriminant validity of the TOS has been ensured according to the Fornell and Larcker (1981) criterion (see Table 2).

*Dependent (Predicted) Variable: Teacher Professional Collaboration Scale (TPCS).* The professional collaboration levels of the participants were measured using TPCS, which is an 8-item (item example: at this school, we share the new ideas and methods that we learn with our colleagues) subscale of the Teacher Leadership Culture Scale developed by Demir (2014). TPCS is a 5-point Likert-type scale in which each item has response options varying from 1 (never) to 5 (always). The CFA of the scale conducted in this study showed high goodness-of-fit values (*χ*^2^/df = 42.293/20 = 2.11; RMSEA = 0.045, SRMR = 0.030, CFI = 0.97, and TLI = 0.95). The Cronbach’s alpha and CR coefficients of the scale were 0.75, while its AVE value was 0.53. Additionally, since the square root of the AVE value of the TPCS (√AVE = 0.73) is higher than the correlations with the scales of DL (*r* = 0.38), TE (*r* = 0.52), and TO (*r* = 0.54), the discriminant validity of the TPCS has been ensured according to the Fornell and Larcker (1981) criterion (see Table 2).

*Control Variables:* Previous studies provided evidence that the demographic variables of participants could affect the professional collaboration characteristics of teachers (de Vries et al., 2013; Shaukat, 2012). For this reason, the gender, education status, and professional experience variables of the participants were analyzed as control variables in this study.

### 2.3 Data analysis

The data collected in the study were analyzed using the Mplus 8.3 (Muthén and Muthén, 2017) and SPSS PROCESS Macro 3.5.3 (Hayes, 2022) statistical package programs. Hypotheses H1, H2, H3, and H4 of the study were tested using Model 4 among PROCESS Macro models, while hypotheses H5 and H6 were tested using Model 8. Before the analyses, data loss values, outlier values, and univariate and multivariate normality assumptions were checked. Because the data collection process took place online, no missing data were encountered. The univariate normal distribution assumptions of the data were tested using skewness and kurtosis values. Skewness and kurtosis values between −1.5 and +1.5 indicate that univariate normality assumptions are met (Tabachnick and Fidell, 2013). The outlier values of the variables were evaluated based on the Mahalanobis distance. The data collected from 16 participants that contained outlier values with Mahalanobis distance values greater than the chi-squared threshold value in a 99.7% confidence interval were excluded from the analyses. Additionally, it was seen that the points on the Q-Q plots were close to the 45-degree reference line. On the other hand, the multivariate normality of the data used in the study was also tested based on Mardia’s multivariate skewness and kurtosis coefficients. The analysis revealed that the *p*-value obtained for skewness was above 0.05, while the kurtosis coefficient fell within the ±1.96 range. These results indicate that the data set meets the multivariate normality assumption (Korkmaz et al., 2014).

To check whether there was a multicollinearity problem between the independent variables of the study, the Pearson product-moments correlation analysis, variance inflation factor (VIF), tolerance, and condition index (CI) values were used. The Durbin-Watson statistic was used to determine whether the error terms were independent (no autocorrelations). According to the results of these tests, the correlation values between the independent variables were smaller than 0.90, the VIF values were smaller than 5, the CI values were smaller than 30, the tolerance values were greater than 0.20, and the Durbin-Watson statistic was close to 2. These results showed that there was no multicollinearity or autocorrelation problem in the data (Field, 2013).

Collecting all data from the same participants and within the same time frame via self-reporting may raise the risk of Common Method Bias (MacKenzie and Podsakoff, 2012). Both procedural and statistical measures were taken to minimize this risk. Procedurally, the scale form instructions emphasized that participants’ identity information would be kept confidential, that responses would be used solely for scientific purposes, and that there were no right or wrong answers, in an effort to reduce the social desirability effect (Podsakoff et al., 2003). In this context Harman’s (1967) one factor test was used to test the common method bias of the study. The results revealed that the first factor explained less than 50% of the total variance (42.88%). Similarly, a confirmatory factor analysis-based supplementary analysis was conducted to assess the effect of common method variance (CMV) in the study. In this regard, a single-factor model, in which all variables loaded onto a single factor, was first tested; it was then compared with the scale’s original multi-factor structure. When examining the fit indices, it was observed that the single-factor model showed a poor fit (*χ*^2^/df = 8.07; RMSEA = 0.15; SRMR = 0.13; CFI = 0.58; TLI = 0.61). These results indicate that common method variance did not have a significant effect on the data and that the explanatory power of a single factor was weak (Fuller et al., 2016). The data of the study were also collected according to the ordering of the dependent, independent, moderator, and mediator variables. This way, the risk of the responses of the participants being caused by the scale format was minimized by changing the ordering of the variables of the study. The direct and indirect effects of the exogenous variables on the endogenous variable were examined using the bootstrap method. In the analyses, 95% confidence intervals for the effects estimated with 5,000 iterations were taken into account. In cases where confidence intervals do not contain the value zero (0), it is assumed that the direct or indirect effects are statistically significant (Hayes, 2022). The model fit degrees of the measurement instruments were interpreted based on their chi-squared/degrees of freedom (*χ*^2^/df), RMSEA, SRMR, CFI, and TLI values. Among goodness-of-fit indices, *χ*^2^/df values smaller than 5, SRMR values smaller than 0.05, RMSEA values smaller than 0.08, and CFI and TLI values greater than 0.90 indicate that the established model is confirmed (Hu and Bentler, 1999; Kline, 2005). The conditional indirect effects of DL on TPC mediated by TE were also examined based on low (−1 SD), medium (mean), and high (+1 SD) levels of TO.

### 2.4 Level of analysis and measurement approach

In this study, distributed leadership, which is inherently a school-level variable, was examined through teachers’ individual perceptions. The focus of the research is to reveal the psychological effect of the sharing of leadership functions on teachers’ internal states (TE and TO), rather than the structural characteristics of the school. According to social cognitive theory, the main factor shaping an individual’s attitudes and behaviors is not so much objective reality as how the individual perceives that reality (Bandura, 1986). Therefore, analyses were conducted at the individual level to measure how leadership practices and the school organizational structure were experienced by teachers. Although the scale used assesses the distribution of leadership, the analysis of the data set reflects the teachers’ subjective assessments.

## 3 Results

### 3.1 Descriptive statistics and correlation analysis results

Table 2 presents the descriptive statistics of all variables included in the study, AVE and √AVE values, reliability coefficients, and Pearson’s correlation analysis results. Cronbach’s alpha and CR coefficients higher than 0.70, AVE values higher than 0.50, correlations between the √AVE values of the scales and other scales and CR coefficients higher than AVE values indicate that convergent validity is provided, and the scales have sufficient reliability and validity (Fornell and Larcker, 1981; Fraenkel et al., 2012). Additionally, as shown in Table 2, while the DL (*M* = 4.26, *SD* = 0.76) and TO (*M* = 4.07, *SD* = 0.77) levels of the participants were above-average, their TE (*M* = 3.41, *SD* = 1.08) and TPC (*M* = 3.29, *SD* = 0.73) levels were moderate. Based on their SD values, TE (*SD* = 1.08) showed greater variance in comparison to DL (*SD* = 0.76), TO (*SD* = 0.77), and TPC (*SD* = 0.73). Positive and statistically significant relationships were identified between DL and TE (*r* = 0.39; *p* < 0.01), TO (*r* = 0.50; *p* < 0.01), and TPC (*r* = 0.38; *p* < 0.01), between TE and TO (*r* = 0.66; *p* < 0.01) and TPC (*r* = 0.52; *p* < 0.01), and between TO and TPC (*r* = 0.54; *p* < 0.01).

### 3.2 Hypothesis test results

**Figure 2:**
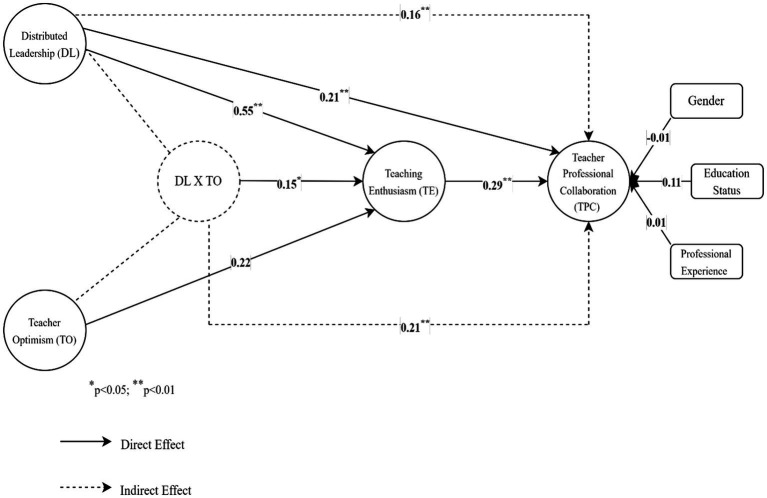
Analysis results of the moderated mediation test.

The variables included in the study were analyzed at three stages: the indirect effect of DL on TPC, the moderator role of TO in the effect of DL on TE, and the moderated mediation effect of TE in this relationship. As seen in Table 3 and Figure 2, DL had significant direct effects on TPC (*b* = 0.21, 95% CI [0.13, 0.28]) and TE (*b* = 0.55, 95% CI [0.44, 0.66]). These results supported H1 and H2. The direct effect of TE on TPC was also significant (*b* = 0.29, 95% CI [0.24, 0.34]), which supported H3. The analysis results revealed that the indirect effect of DL on TPC mediated by TE was significant and positive (*b* = 0.16, 95% CI [0.12, 0.20]). Thus, H4 was supported.

**Table 3:** Unstandardized coefficients of direct, mediator/indirect, moderator, and moderated mediator effects (*n* = 547).

Variables	Direct effects	Mediating effect	Moderator effect	Moderated mediator effect
DL → TPC	0.21^**^ [0.13, 0.28]^***^			
DL → TE	0.55^**^ [0.44, 0.66]^***^			
TE → TPC	0.29^**^ [0.24, 0.34]^***^			
DL → TE → TPC		0.16^**^ [0.12, 0.20]^***^		
(DL × TO) → TE			0.15^*^ [0.02, 0.27]^***^	
(DL × TO) → TE → TPC				0.21^**^ [0.12, 0.30]^***^
Moderated mediation index	0.02 [0.01, 0.05]^***^			

*p < 0.05; **p < 0.01; ***%95 confidence interval (CI); DL, distributed leadership; TO, teacher optimism; TE, teaching enthusiasm; TPC, teacher professional collaboration.

**Figure 3:**
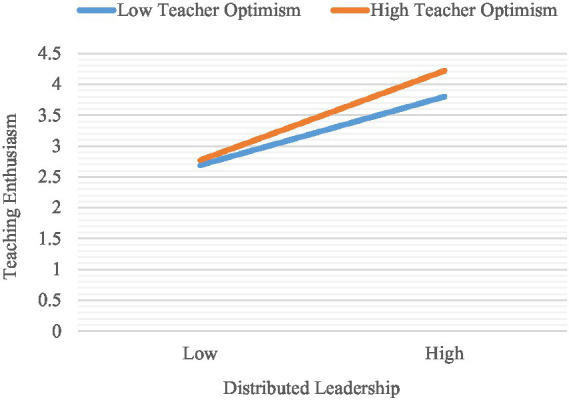
Plot of the moderator effect of teacher optimism in the relationship between distributed leadership and teaching enthusiasm.

The moderator role of TO in the effect of DL on TE was found to be positive and significant (*b* = 0.15, 95% CI [0.02, 0.27]) (Table 3; Figure 2). The slope of the simple regression curve was calculated to determine whether the effect of DL on TE varied significantly depending on TO (Figure 3). As seen in Table 4, in cases where TO was moderate (*b* = 0.17, *SE* = 0.06, 95% CI [0.05, 0.28]) and high (*b* = 0.28, *SE* = 0.09, 95% CI [0.10, 0.46]) the effects of DL on TE were stronger. In cases where TO was low, the effects of DL on TE were weak and statistically insignificant (*b* = 0.06, *SE* = 0.06, 95% CI [−0.06, 0.17]) (Table 4). Therefore, H5 was supported.

**Table 4:** Effects of distributed leadership on teaching enthusiasm linked with levels of teacher optimism.

	Estimate	SE (Boot)	95% CI	95% CI
−1 Std. deviation	0.06	0.06	−0.06	0.17
Medium (mean)	0.17	0.06	0.05	0.28
+1 Std. deviation	0.28	0.09	0.10	0.46

Finally, the effects of TO on the mediation model in cases where it was low (−1 SD), medium, and high (+1 SD) were tested. In this regard, it has been determined that TO has a moderating effect on DL’s impact on TPC through TE (*b* = 0.21, 95% CI [0.12, 0.30]). Similarly, the moderated mediation index was statistically significant (moderated mediation index = 0.02 [0.01, 0.05]) (Table 3; Figure 2). In other words, in cases where TO levels were medium (*b* = 0.03, *SE* = 0.01, 95% CI [0.01, 0.05]) and high (*b* = 0.05, *SE* = 0.02, 95% CI [0.02, 0.08]), the indirect effects of DL on TPC mediated by TE were stronger. In cases where TO was low (*b* = 0.01, *SE* = 0.01, 95% CI [−0.01, 0.03]), the indirect effects of DL on TPC mediated by TE were weaker and statistically insignificant (Table 5). These results supported H6. On the other hand, the control variables of gender (*b* = −0.01, 95% CI [−0.11, 0.09]), education status (*b* = 0.11, 95% CI [−0.02, 0.25]), and professional experience (*b* = 0.01, 95% CI [−0.01, 0.01]) were found to have no significant relationship to TPC in the moderated mediation model.

**Table 5:** Effects of distributed leadership on professional collaboration of teachers mediated by teaching enthusiasm linked with levels of teacher optimism.

	Estimate	SE (Boot)	95% CI	95% CI
−1 Std. deviation	0.01	0.01	−0.01	0.03
Medium (mean)	0.03	0.01	0.01	0.05
+1 Std. deviation	0.05	0.02	0.02	0.08

## 4 Discussion and conclusion

The study focused on identifying the previously undetermined complex relationships between distributive leadership, teacher professional collaboration, teaching enthusiasm, and teacher optimism, which were found in the literature to be associated with improving the quality of education, enhancing teaching, and achieving successful student outcomes. In this regard, the study discovered some very important direct and indirect relationships.

The first hypothesis (H1) proposed that there is a direct and positive relationship between DL and TPC. The results confirmed this hypothesis by showing that when school principals involved teachers in school-related decisions and considered their opinions and suggestions in solving problems, teachers collaborated more with their colleagues in teaching practices and solved problems that could arise inside or outside the classroom together. It is an expected outcome that the DL approach (Liden et al., 2008), which views leadership as an interactive and collective practice, would be related to TPC, which explains the collective efforts of teachers. According to the conservation of resources theory, TPC provides teachers with a social resource (Hobfoll, 2002). DL’s maintenance of leadership through the interaction of numerous school stakeholders and its encouragement of teachers’ TPC increases their social resources and expands their capacities. This finding is consistent with previous studies that examined TPC in relation to a leadership approach that DL practices throughout the school (Çoban and Atasoy, 2020; Lin, 2022).

The second hypothesis (H2) predicted a significant relationship between DL and TE. The findings revealed that when principals fostered a democratic environment in the school by DL roles, teachers approached their teaching responsibilities with greater enthusiasm. This confirmed the meaningful and direct relationship between DL and TE, thereby supporting the second hypothesis. This research explains that in Turkish schools, characterized by collectivist traits and a centralized structure (Hofstede, 1980), when school principals relax the centralized structure, removing leadership from being a hierarchical mechanism and distributing leadership roles throughout the school, this fuels teachers’ TE. This result of the study is consistent with the research base showing that the DL approach increases teachers’ TE’s (Doğan, 2025), commitment (Lin, 2022) and leads them to use more innovative practices in their profession (Büyükgöze et al., 2024; Lin, 2022). The positive effects of teachers’ teaching enthusiasm on professional commitment and student achievement are also important and noteworthy in the context of the Turkish education system.

The third hypothesis of the study (H3) suggested that TE is related to TPC, enabling the examination of a different direct relationship. The analysis results supported this assumption by showing a positive and direct relationship between TE and TPC. This finding of the study showed that when teachers can share new ideas, methods, and teaching strategies with their colleagues and examine each other’s practices, they derive more pleasure from teaching and conduct their lessons with greater enthusiasm. This confirmed the third hypothesis and made an important contribution to an uncertain area in the literature. Kunter et al. (2011) state that TE is related to teachers’ individual characteristics. Therefore, it is expected that teachers who are enthusiastic about teaching their subject knowledge will also be willing to collaborate with their colleagues. This is also supported by the Broaden-and-Build Theory, which reflects the perspective of positive psychology. According to this theory, positive emotions such as enthusiasm broaden individuals’ repertoire of actions, such as collaborating with colleagues. A teacher with TE also strengthens their social bonds with colleagues and becomes more willing to collaborate (Fredrickson, 2004). This finding supports previous research showing that teachers with high TE also have high feelings of success, experience lower levels of burnout (Kasalak and Dağyar, 2020), and can increase their students’ motivation and success by spreading their enthusiasm to them (Burić, 2019).

The results of the fourth hypothesis (H4) showed that TE played a mediating role in the relationship between their principals’ DL and TPC. This finding implies that principals delegating more of their leadership roles to teachers, allowing teachers to have more say in school management, may lead to teachers being more enthusiastic and to collaborate more with their colleagues when teaching their subject matter. TE describes a teacher’s flow experience. A teacher who has teaching enthusiasm has a high level of commitment to their profession. This commitment and inner passion can facilitate collaboration among teachers. School administrators who distribute leadership also facilitate a dynamic interaction between the collective awareness they create in their schools and the opportunities they offer teachers and the teachers’ individual talents (Csikszentmihalyi et al., 2018). Thus, teachers who are more involved in school management may conduct their lessons with enthusiasm and be more willing to collaborate with their colleagues. The findings are consistent with Özdemir et al. (2022), which showed the mediating role of teacher enthusiasm between leadership and teaching practices, and provide new evidence on the relationship between school leadership and professional collaboration by considering teaching enthusiasm as a mediating variable (Ma and Marion, 2024; Meyer et al., 2020).

The results of the fifth hypothesis (H5) have made an original contribution to the literature by showing that TO strengthens the relationship between DL and TE. It has shown that when principals in Turkish schools involve teachers in school management, teachers can deliver their lessons with greater enthusiasm, and that this can be even stronger when teachers maintain their optimism despite adversity. Optimism is one of the important internal resources that individuals possess (Cozzarelli, 1993). This result may be related to optimistic individuals having a more positive outlook and possessing greater resilience and coping skills in the face of adversity (Scheier and Carver, 1992). The findings also make an important contribution to the literature on what is important in supporting teachers’ enthusiasm (Kouhsari et al., 2023; Sheppard et al., 2010). In other words, this finding of the research may encourage proactive practices in the policies to be determined so that teachers can deliver more enthusiastic lessons.

The sixth hypothesis (H6) demonstrated that TO moderates the relationship between DL and TPC through TE. In practice, this means that optimistic teachers, when they feel valued as members of the school community with the support of the principal, will teach with greater enthusiasm and exert more effort for student success. Furthermore, this finding is similar to the finding identified in the study by Kouhsari et al. (2023) that teacher enthusiasm mediates the relationship between collective teacher innovation emerging from teacher collaboration and school climate. The literature points to the difficulties of achieving school goals in centralized education systems (Thien, 2019). Therefore, the results of the current study are particularly important for Turkish schools with a centralized education system in terms of relaxing centralization and implementing school democracy. The findings suggest that in countries with similar cultural characteristics (e.g., Azerbaijan, Iran, Iraq) and centralized education systems (e.g., France), as Turkey, principals can increase TE and cooperation by leveraging teachers’ optimism to spread leadership. Therefore, this research also responds to Keller et al. (2016)‘s call for future studies to reveal the relationship between teaching enthusiasm and other emotional experiences with hypotheses H5 and H6.

The results obtained in this study should be evaluated not only in terms of statistical significance (*p* < 0.05) but also in terms of their contribution to educational management practice and magnitude of effect. According to the analysis results, the research model incorporating the regulatory role of TO explains approximately 38% of the total variance in TPC behaviors. Considering the criteria established by Cohen (1988) for social science research, this ratio indicates a high level of effect size. This result proves that DL practices and teachers’ emotional states are among the most powerful determinants shaping the culture of collaboration in schools. Furthermore, when TE, the model’s mediating mechanism, is considered, the explanatory power of the model is seen to increase even further. DL, teacher optimism, and the interaction of these two variables explain 45% of the variance in teachers’ enthusiasm levels. This ratio, which is considered quite high in terms of workforce motivation, shows that school administrators’ ways of distributing leadership, combined with teachers’ levels of optimism, determine almost half of their enthusiasm for the profession. Finally, the significance of the moderated mediation index (Index = 0.02; 95% CI [0.01, 0.05]) confirms that this practical effect is conditional. In other words, school principals’ DL practices have a much stronger effect on increasing collaboration, particularly among teachers with high levels of optimism. This result reveals that it is not enough for school administrators to simply distribute tasks to increase collaboration; it is also a practical necessity to create a climate that strengthens teachers’ psychological capital.

In conclusion, this study examined school administrators’ leadership practices, teachers’ collaborative behaviors, and emotional characteristics such as optimism and enthusiasm for teaching through the lens of distributive leadership theory and the positive psychology approach. The leadership approach, which distributes positive emotions such as optimism and enthusiasm, which are at the core of positive psychology, and leadership roles to all teachers in the school, can help increase collaboration among teachers and positive workplace behaviors (Cameron et al., 2003; Carr, 2019). This research indicates that teachers being enthusiastic while teaching and taking an active role in school management may enable them to be more collaborative in fulfilling these roles and other teaching roles. This is because enthusiastic teachers are willing to make an effort, exhibit proactive behaviors, and develop their own competencies while performing their profession. Enthusiasm is contagious by nature, and enthusiastic teachers can positively influence the school climate (Burić and Moè, 2020; Wenström et al., 2018). Therefore, teachers’ enthusiasm for teaching can function as an emotional mechanism that connects teachers’ collaboration with a leadership approach that distributes leadership throughout the school. Moreover, when teachers are more optimistic, they trust school administrators’ leadership practices more, participate more in management, and can teach more collaboratively and enthusiastically (Hoy and Tarter, 2011). Thus, teachers’ positive psychological characteristics, such as enthusiasm and optimism, and school administrators’ DL characteristics can contribute to a collaborative school culture, a positive school climate, teachers’ increased interest in their profession, and increased school commitment and well-being (Yang, 2022; Li et al., 2016; Li et al., 2024; Wenström et al., 2018).

### 4.1 Theoretical implications

The research expands the literature showing that school administrators’ leadership approaches affect teachers’ emotions. By pointing to the important role of teachers’ TE and TO in the school’s organizational functioning, it advances our understanding of the relationship between DL and TPC. The research showed that DL affected teachers’ enthusiasm for teaching and that this effect also encouraged teachers to behave more collaboratively with their colleagues. Furthermore, it has been theoretically validated and empirically proven that this indirect effect is strengthened in situations where TO is high. In addition, the research has demonstrated the positive relationship between TE and TPC, another area lacking in the research literature, and has identified the moderating role of TO in the relationship between DL and TE. The results of the study can shed light on subsequent research as an important source of information on these uncertain areas. This research shows that in a centralized education system, principals’ DL characteristics make teachers’ TE more active. This indicates that when school principals use DL to loosen centralization, teachers may become more enthusiastic about teaching. Enthusiastic teachers become more willing to collaborate with their colleagues. Moreover, this mechanism is shaped by teachers’ optimism. As a personal resource, optimism has the potential to guide teachers’ consistent and goal-oriented actions (Scheier and Carver, 1992). When teachers’ optimism is high, the power of this mechanism increases. Thus, the study also provides important implications for policymakers and practitioners by facilitating a better theoretical understanding of how DL affects teacher collaboration, enabling them to determine policies that strengthen the distributive leadership characteristics of school principals and enable teachers to maintain their optimism.

### 4.2 Practical results and recommendations

According to the findings of the study, school principals distributing their leadership roles to teachers and the enthusiasm and optimism possessed by teachers contribute to the formation of a strong culture of cooperation within the school. This situation increases teachers’ commitment to their profession and can also strengthen solidarity, reflecting a culture of “collective work.” School administrators’ leadership practices are significantly related to teachers’ emotions (Çevik and Doğan, 2025). School administrators’ leadership behaviors are decisive in teachers experiencing negative emotions such as burnout and stress. Therefore, school administrators can adopt the DL approach to increase teachers’ motivation by distributing leadership roles more among teachers. They can encourage teachers to participate in school decisions and express their opinions openly. By trusting teachers’ abilities, appreciating their dedicated work, and supporting their professional development, they can ensure that their motivation remains high (Lambersky, 2016). Furthermore, school administrators can reduce their own workload by distributing leadership roles. They can then use the time they gain to organize different activities that teachers can collaborate on, thereby increasing the school’s capacity (Kaya et al., 2024; OECD, 2014). Teachers who participate more in management may approach teaching processes with greater enthusiasm. This positively affects student achievement by improving the quality of education. TO stands out as a key element supporting this process. Teachers who collaborate with their colleagues can discover ways to help their students learn better together (Slavit et al., 2011).

Teachers can create an educational environment that satisfies students with their enthusiastic teaching and prevent negative situations that may arise with their optimism (Öngel and Tabancalı, 2022). It is known that cultural differences affect teachers’ enthusiasm for teaching (Zhang and Ye, 2024). Therefore, school administrators may be advised to use incentives that are sensitive to teachers’ needs and culture in order to ignite their enthusiasm. Additionally, administrators should manage school life and daily school routines in a way that supports teachers’ enthusiasm and optimism. For example, they can minimize tedious paperwork that takes up a significant portion of teachers’ time. This can help teachers maintain their commitment to their profession and support their efforts to help students learn (Dannetta, 2002). By instilling feelings of optimism and enthusiasm about the future in their students, teachers can both increase their success and help them develop their personalities in a positive direction (Kashy-Rosenbaum et al., 2018; Seligman, 2019). This also applies to Turkish teachers. In this context, it can be suggested that Turkish policymakers develop policies that increase teachers’ enthusiasm by relaxing the centralized structure and allowing principals to share leadership. This requires policymakers to address normative and organizational challenges and implement changes at the school level (Doğuş et al., 2025). Furthermore, based on the premise that optimism is a learnable emotional skill (Seligman, 2020), policy regulations aimed at creating workplace environments that enhance teachers’ subjective well-being and welfare could also be beneficial.

### 4.3 Limitations of the study and recommendations for future research

The study has several limitations. Since the study was designed as a cross-sectional study within a single time frame, it cannot prove causal relationships between variables. Therefore, causal relationships between the study variables can be examined through longitudinal or experimental studies. In other words, although the relationship between DL and TE has been theoretically addressed in a cause-and-effect context, the possibility that this relationship may be reciprocal, or cyclical should not be overlooked. Although the study participants were sufficient in terms of sample size, they may not fully represent the diversity of all teachers in Turkey. Repeating the study with a larger sample group may yield different results. Since the study data were collected online, the distribution of participating teachers at the school level could not be determined. Statistical and procedural measures were taken to eliminate common method bias in the study. However, measuring the study variables from various participant groups, such as both teachers and school leaders, could reduce common method bias and increase the validity of the study. Similarly, only scales were used as data collection tools in the study. The research results could be examined in greater depth using data collection tools such as interviews and observations, in line with qualitative or mixed methods approaches. The fact that the research data was collected from schools in Turkey, where the education system is bureaucratic and centralized, may prevent the results from being generalized to Western countries where teacher autonomy is high. A similar study could be replicated in Western countries and comparative analyses could be conducted.

## Data Availability

The raw data supporting the conclusions of this article will be made available by the authors, without undue reservation.
